# Minimally invasive versus open pancreatoduodenectomy (LEOPARD-2): study protocol for a randomized controlled trial

**DOI:** 10.1186/s13063-017-2423-4

**Published:** 2018-01-03

**Authors:** Thijs de Rooij, Jony van Hilst, Koop Bosscha, Marcel G. Dijkgraaf, Michael F. Gerhards, Bas Groot Koerkamp, Jeroen Hagendoorn, Ignace H. de Hingh, Tom M. Karsten, Daan J. Lips, Misha D. Luyer, I. Quintus Molenaar, Hjalmar C. van Santvoort, T. C. Khé Tran, Olivier R. Busch, Sebastiaan Festen, Marc G. Besselink, for the Dutch Pancreatic Cancer Group

**Affiliations:** 1 Department of Surgery, Academic Medical Center, Cancer Center Amsterdam, PO Box 22660, 1100 DD Amsterdam, The Netherlands; 2 Department of Surgery, Jeroen Bosch Hospital, PO Box 90153, 5200 ME Den Bosch, The Netherlands; 3 Clinical Research Unit, Academic Medical Center, PO Box 22660, 1100 DD Amsterdam, The Netherlands; 4 Department of Surgery, Onze Lieve Vrouwe Gasthuis, PO Box 95500, 1090 HM Amsterdam, The Netherlands; 5 Department of Surgery, Erasmus University Medical Center, PO Box 2040, 3000 CA Rotterdam, The Netherlands; 6 Department of Surgery, University Medical Center Utrecht, PO Box 85 500, 3508 GA Utrecht, The Netherlands; 7 Department of Surgery, Catharina Hospital, PO Box 1350, 5602 ZA Eindhoven, The Netherlands

**Keywords:** Minimally invasive, Laparoscopic, Pancreatoduodenectomy, Whipple, Robot-assisted

## Abstract

**Background:**

Data from observational studies suggest that minimally invasive pancreatoduodenectomy (MIPD) is superior to open pancreatoduodenectomy regarding intraoperative blood loss, postoperative morbidity, and length of hospital stay, without increasing total costs. However, several case-matched studies failed to demonstrate superiority of MIPD, and large registry studies from the USA even suggested increased mortality for MIPDs performed in low-volume (<10 MIPDs annually) centers. Randomized controlled multicenter trials are lacking but clearly required. We hypothesize that time to functional recovery is shorter after MIPD compared with open pancreatoduodenectomy, even in an enhanced recovery setting.

**Methods/design:**

LEOPARD-2 is a randomized controlled, parallel-group, patient-blinded, multicenter, phase 2/3, superiority trial in centers that completed the Dutch Pancreatic Cancer Group LAELAPS-2 training program for laparoscopic pancreatoduodenectomy or LAELAPS-3 training program for robot-assisted pancreatoduodenectomy and have performed ≥ 20 MIPDs. A total of 136 patients with symptomatic benign, premalignant, or malignant disease will be randomly assigned to undergo minimally invasive or open pancreatoduodenectomy in an enhanced recovery setting. After the first 40 patients (phase 2), the data safety monitoring board will assess safety outcomes (not blinded for treatment allocation) and decide on continuation to phase 3. Patients from phase 2 will then be included in phase 3. The primary outcome measure is time (days) to functional recovery. All patients will be blinded for the surgical approach, at least until postoperative day 5, but preferably until functional recovery has been attained. Secondary outcome measures are operative and postoperative outcomes, including clinically relevant complications, mortality, quality of life, and costs.

**Discussion:**

The LEOPARD-2 trial is designed to assess whether MIPD reduces time to functional recovery, as compared with open pancreatoduodenectomy in an enhanced recovery setting.

**Trial registration:**

Netherlands Trial Register, NTR5689. Registered on 2 March 2016.

**Electronic supplementary material:**

The online version of this article (doi:10.1186/s13063-017-2423-4) contains supplementary material, which is available to authorized users.

## Background

Pancreatoduodenectomy is the only potential curative treatment option for periampullary cancer and premalignant tumors. In 1994, minimally invasive pancreatoduodenectomy (MIPD) was introduced [1]. After a slow introduction, the use of MIPD has increased rapidly in recent years. Several cohort studies have suggested that MIPD is safe and feasible when performed by experienced surgeons in high-volume centers [2, 3]. However, the benefits of MIPD in real-life clinical practice remain unclear. On the one hand, meta-analysis of cohort studies showed that MIPD is associated with decreased intraoperative blood loss (weighted mean difference, −384 mL; *P* < 0.01), less delayed gastric emptying (odds ratio, 0.7; *P* < 0.01) and decreased length of hospital stay (weighted mean difference, −3 days; *P* < 0.01) compared with open pancreatoduodenectomy. On the other hand, MIPD increases operative time significantly and registry studies have expressed concerns on its safety [4]. Compared with open pancreatoduodenectomy, mortality might be doubled after MIPD in low-volume centers performing fewer than 10 MIPD procedures annually (8% versus 3%; *P* < 0.01) [4]. These studies raise concerns about the feasibility and generalizability of MIPD and clearly demonstrate the need for a randomized controlled trial, especially in this era when minimally invasive pancreatic surgery is gaining popularity worldwide [2, 5].

The cost-effectiveness and quality of life associated with MIPD have currently only been reported in small observational studies [6]. These studies reported higher operative costs of MIPD, which were compensated by lower postoperative costs because of shorter hospital stay [3]. However, the limited sample sizes of these studies do not allow reliable conclusions. Moreover, outcomes of open pancreatoduodenectomy have also improved in recent years with enhanced recovery strategies leading to shorter postoperative hospital stay [7]. These parameters should therefore be assessed in a multicenter randomized trial using an enhanced recovery setting for both MIPD and open pancreatoduodenectomy.

In the Netherlands, a platform for such a multicenter randomized trial has been created by the Dutch Pancreatic Cancer Group (DPCG), a nationwide multidisciplinary group including all 17 centers performing pancreatic surgery in the Netherlands. The DPCG followed the internationally accepted Idea, Development, Exploration, Assessment, and Long-term (IDEAL) study framework for surgical innovation for the implementation of minimally invasive pancreatic surgery in the Netherlands [8–10]. Since 2014, the DPCG successfully implemented minimally invasive distal pancreatectomy in all Dutch pancreatic centers within the LAELAPS (longitudinal assessment and realization of laparoscopic pancreatic surgery) project [11]. Thereafter, the DPCG initiated a similar nationwide training program for laparoscopic pancreatoduodenectomy (the LAELAPS-2 project) [12]. This program included detailed technique description, video training, and proctoring sessions (on-site and off-site) by (inter)national experts. Analysis of outcomes of 114 MIPDs performed within LAELAPS-2, in the first four DPCG centers that performed >20 MIPDs, showed an 11% conversion rate, a 43% Clavien–Dindo III or higher complication rate, and a 4% complication-related 90-day mortality [12]. Hereafter, a structured training program in robot-assisted pancreatoduodenectomy using the Pittsburgh approach (the LAELAPS-3 project) was implemented [13]. Based on these outcomes and the IDEAL study framework [8–10], it was decided that a randomized controlled trial was indicated in those centers that performed at least 20 MIPDs.

The LEOPARD-2 trial follows the LEOPARD trial on minimally invasive versus open distal pancreatectomy [14] but has a phase 2/3 design to assess patient safety more closely. The aim of the LEOPARD-2 trial is to compare time to functional recovery after surgery, complications, quality of life, and costs after MIPD versus open pancreatoduodenectomy, within an enhanced recovery setting.

## Methods

### Design

The LEOPARD-2 trial is a randomized controlled, parallel-group, patient-blinded, multicenter, phase 2/3, superiority trial investigating the safety and effectiveness of MIPD versus open pancreatoduodenectomy, in an enhanced recovery setting at high-volume (≥20 pancreatoduodenectomies annually) pancreatic centers who completed a dedicated MIPD training program. Eligible patients will be randomized in a 1:1 ratio to either minimally invasive or open pancreatoduodenectomy.

### Trial population

All adult patients with an indication for elective pancreatoduodenectomy because of malignant, premalignant, or symptomatic benign disease in the pancreatic and periampullary region will be screened for eligibility.

### Inclusion criteria

Inclusion criteria are as follows:

- Age at least 18 years
- Indication for elective pancreatoduodenectomy
- Both minimally invasive and open pancreatoduodenectomy feasible, according to the surgical team (based on computed tomography performed a maximum of 4 weeks before surgery)
- Fit to undergo pancreatoduodenectomy, according to the surgical and anesthesiological team

### Exclusion criteria

Exclusion criteria are as follows:

- Tumor involvement of portal vein, superior mesenteric vein, superior mesenteric artery, or hepatic artery (based on computed tomography performed a maximum of 4 weeks before surgery)
- Body mass index > 35 kg/m^2^
- Neo-adjuvant pancreatic radiotherapy
- Second cancer requiring resection during the same procedure
- Pregnancy
- Participation in another study with interference of study outcomes

### Randomization

**Fig. 1:**
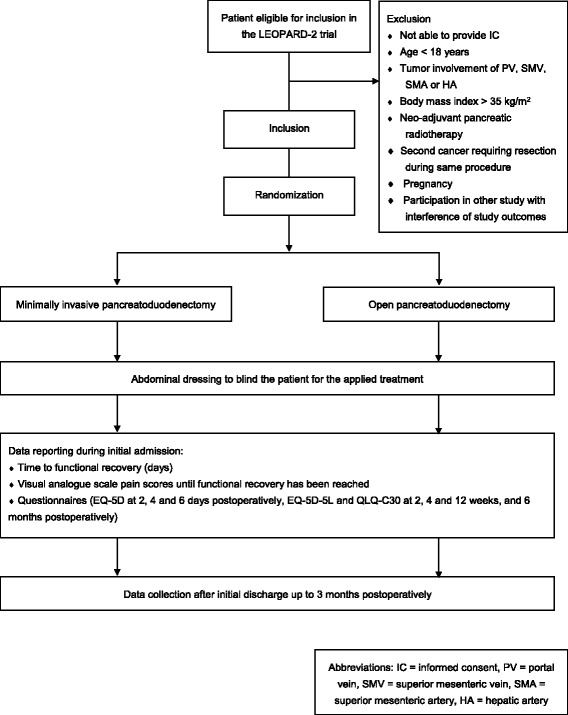
LEOPARD-2 trial flow diagram according to SPIRIT [36]. EQ-5D-5 L, Euro-QoL five health dimensions questionnaire; HA, hepatic artery; IC, informed consent; PV, portal vein; QLQ-C30, Quality of life questionnaire including 30 questions; SMA, superior mesenteric artery; SMV, superior mesenteric vein

Eligible patients will be recruited at the outpatient clinic. Written informed consent must be provided before inclusion. Included patients will be randomized centrally by the study coordinators (TdR and JvH) using an online randomization module (ALEA, Clinical Research Unit, Academic Medical Center, Amsterdam, the Netherlands) in a 1:1 ratio between MIPD and open pancreatoduodenectomy (Fig. 1). Randomization will be stratified by center to balance differences in surgical procedure and general treatment regimen and by the preoperative probability of developing a postoperative pancreatic fistula: high risk (i.e. pancreatic duct diameter < 3 mm or body mass index > 25 kg/m^2^) versus low risk (i.e. i.e. pancreatic duct diameter ≥ 3 mm and body mass index ≤ 25 kg/m^2^). Stratification based on intraoperative patient characteristics was considered impossible. Permuted-block randomization will be used to provide treatment allocation in equal proportions, with block sizes ranging from 2 to 6, subject to random variation. This will be concealed to all investigators involved in the trial.

### Intervention: minimally invasive pancreatoduodenectomy

Here we describe the standard operative technique. Small variations according to the surgeon’s preference, such as a different order of steps or pancreatic anastomosis technique, are allowed, but must be recorded in the case record form. Both laparoscopic surgery and robot-assisted surgery, e.g. using the da Vinci® Surgical System, are allowed as both are considered equivalent methods of MIPD.

All procedures are performed by two trained gastrointestinal surgeons (see section on quality and safety). Patients are under general anesthesia; epidural is not mandatory. The patient is placed in a supine position on a bean bag. The right arm is placed along the body and the left arm in 90° abduction. The suprapubic region is kept free for a Pfannenstiel incision. Trocars are placed in a soft semicircular fashion: subumbilical (12 mm trocar), left and right to the umbilicus (two 12 mm trocars), 4 cm subcostal (two 5 mm trocars), with an optional 5 mm subxiphoïdal trocar for liver retraction. Diagnostic laparoscopy is performed to rule out metastasis. The cystic duct and artery are transected, the gallbladder is partly mobilized and sutured to the anterior abdominal wall for liver retraction. Likewise, the round ligament is retracted to the anterior abdominal wall, with either a suture or retractor. The lesser sac is opened and a Kocher maneuver is performed. The inferior border of the pancreas and the superior mesenteric vein are visualized. The gastro-epiploic artery and vein are transected. Lymph node station 8a is dissected, followed by identification of the hepatic artery, gastroduodenal artery, and portal vein. The gastroduodenal artery is transected between multiple Hem-o-lok clips (Teleflex Medical, Research Triangle Park, NC, USA). A tunnel is created under the pancreas and a 15 cm long vessel loop is passed and secured to itself. The stomach is transected just proximal or distal to the pylorus using an endostapler after temporary removal of the nasogastric tube. The pancreas is transected using diathermia or an ultrasonic or sealing device, but the pancreatic duct is transected sharply with scissors and intubated to ensure that it is open. The first jejunal limb is dissected from the peritoneal reflections, transected using an endostapler, and fully mobilized. The two jejunal ends are fixated using a suture, thus facilitating passing behind the mesenteric root. After retracting the duodenum and jejunum to the right side of the mesenteric root, the duodenum is stretched and the uncinate process is mobilized. First, the superior mesenteric vein and then the superior mesenteric artery branches are mobilized until the pancreatic head is fully dissected. The common hepatic artery is followed up to the right hepatic artery. Retroportal lymph nodes are resected according to the International Study Group on Pancreatic Surgery guidelines [15]. The hepatic duct is tunneled and transected.

After a 15–20 min break, a modified Blumgart pancreatojejunostomy is performed (preferably using 3D laparoscopy) using four 3/0 V-loc barbed sutures (Covidien, New Haven, CT, USA), a 10 cm 6–8 Fr internal pancreatic stent, and four to six duct-to-mucosa sutures (Vicryl (Ethicon Inc., Cincinnati, OH, USA) or Novosyn (B. Braun, Melsungen, Germany). In this way, the jejunal wall is folded around the pancreatic stump. Approximately 5–7 cm distally, on the same jejunal loop, an end-to-side hepaticojejunostomy is performed, either using 10 to 15 12 cm long Vicryl 4/0 interrupted sutures or either two 4/0 V-loc barbed (Covidien, New Haven, CT, USA) or polydioxanone (Ethicon Inc., Cincinnati, OH, USA) running sutures. Through a right subcostal stab incision, a 27 Fr surgical drain is placed through Winslow up to the superior border of the pancreaticojejunostomy. Cholecystectomy is now completed. An antecolic 4–5 cm end-to-side gastrojejunostomy is performed using an endostapler, and closed using a V-loc barbed 3/0 suture (Covidien, New Haven, CT, USA). In the case of a pylorus-preserving pancreatoduodenectomy, an end-to-side duodenojejunostomy is performed using running V-loc barbed 3/0 sutures (Covidien, New Haven, CT, USA). The specimen is extracted through a Pfannenstiel incision, which is subsequently closed in layers. A second surgical drain is placed from the right, at the inferior border of the pancreaticojejunostomy with the tip under the gastrojejunostomy. Alternatively, this second drain may be placed from the left. After hemostatic control, trocars are removed and all 12 mm fascia openings and the skin are closed. Optionally, preperitoneal continuous wound catheters are placed [16]. For robot-assisted pancreatoduodenectomy, the Pittsburgh approach is followed [17].

### Control: open pancreatoduodenectomy

Patients undergo multimodal pain therapy with either an epidural catheter or preperitoneal wound catheters with patient controlled analgesia [16]. Subcostal laparotomy is performed. The steps taken are essentially similar to MIPD but the variation in technique is expected to be larger. Since outcomes of open pancreatoduodenectomy in the Netherlands are good, the surgical technique in the control arm reflects their usual practice; also, the anastomoses will be performed according to local protocol. Procedure variations according to the surgeon’s preference are allowed, but must be recorded in the case record form. A surgical drain can be placed.

### Conversion from MIPD to open pancreatoduodenectomy

Conversion is defined as any MIPD (laparoscopic or robot-assisted) in which a skin incision is used for reasons other than trocar placement or specimen extraction. An unplanned open reconstruction in a patient randomized for MIPD in whom the procedure was started minimally invasively (i.e. procedure with laparoscopic resection and open reconstruction) will be reported as conversion. Patients allocated to MIPD who undergo intraoperative conversion to open pancreatoduodenectomy will still be analyzed in the MIPD group according to intention-to-treat principles. Reasons for conversion are registered and categorized in urgent and non-urgent conversions [18].

### Patient blinding

Patients will be blinded until functional recovery has been reached or at least up to day 5 postoperatively using a large (40 cm × 40 cm) abdominal dressing, which is fixated on the abdomen at the end of the surgical procedure. The quality of blinding is assessed by asking patients whether they think the operation was performed minimally invasive or via an open approach on day 3 and just before removing the abdominal dressing (i.e. when functional recovery has been reached, or on day 5). If earlier inspection is required, attempts are made to maintain patient blinding unless there is a medical reason to remove the dressing (e.g. wound infection). Previous trials in the Netherlands have found this approach feasible [14, 19, 20]. A complete double-blinding, including blinding of medical and nursing ward staff, is not considered feasible.

### General treatment regimen

Postoperative care is similar in both arms per center and based on the enhanced recovery after surgery (ERAS®) principle, which includes pain control, early mobilization, and expansion of oral intake, as desired by the patient. Enhanced recovery is standard practice in participating centers. When patients are functionally recovered, they are essentially medically ready to be discharged. Discharge will however take place after shared decision making between the patient and the local treating team and may be delayed by arrangement of home care (e.g. for drain management).

### Primary outcome measure

The primary outcome measure is time to functional recovery (days), which will be daily assessed by the nurses and ward physicians, and cross-checked by the trial coordinators. Functional recovery is reached when all of the following criteria are met [14]:

- Adequate pain control with oral analgesia only
- Restoration of mobility to an independent level (or to preoperative level if previously impaired)
- Ability to maintain sufficient caloric intake (minimum of 50% required calories)
- Absence of intravenous fluid administration
- No signs of active abdominal infection (In the case of suspected or known abdominal infection this item is met when the patient has no fever, and serum C-reactive protein concentration is decreasing and below 150 mg/L.)

### Secondary outcome measures

The most important secondary outcome measure is the overall rate of Clavien–Dindo III or higher complications. Other secondary outcome measures include total procedure time, operative time (first incision to skin closure), intraoperative blood loss, conversion, complications (pancreatic fistula, delayed gastric emptying, chyle leakage, postoperative bleeding, bile leakage, gastro- or duodenojejunostomy leakage, wound infection), re-intervention (radiologic, endoscopic, or surgical), hospitalization parameters (intensive care unit admission, readmission, length of hospital stay, total of hospitalized days during follow-up), adjuvant chemotherapy in patients with cancer, mortality, and pathology outcomes (resected specimen size, tumor size, histopathological diagnosis, resection margins (in patients with cancer), lymph node retrieval, tumor positive lymph node retrieval (in patients with cancer), perineural and lymphovascular tumor invasion (in patients with cancer), quality of life (EQ-5D-5 L and QLQ-C30 questionnaires), survival, and costs.

### Data collection and follow-up

Baseline characteristics will be collected before randomization using standardized case record forms; this comprises age (years), sex, performance status (Karnofsky score), WHO physical status, American Society of Anesthesiologists physical status, body mass index (kg/m^2^), Malnutrition Universal Screening Tool score, medical history, symptoms and duration of symptoms, conclusion preoperative imaging, tumor size on imaging, TNM (tumor, node, metastasis) stage on imaging, pancreatic duct diameter on imaging (measured at the level of the portal vein), tumor involvement of other organs, preoperative expected diagnosis, preoperative biliary drainage, and use of somatostatin analogs. Operative characteristics include pancreatic texture (soft versus firm), pancreatic duct diameter (mm), and type of anastomoses. Primary and secondary outcome measures will be collected after randomization up to 3 months postoperatively using standardized case record forms by the local treating physicians or the study coordinators. The case record forms and the database will be cross-checked with source data by the study coordinators. Patients will be asked to complete validated questionnaires on postoperative days 2, 4, and 6 (EQ-5D-5 L) and at 2 weeks, 4 weeks, and 12 weeks postoperatively (EQ-5D-5 L and QLQ-C30). Please see Fig. 1. Long-term follow-up results of the LEOPARD-2 trial (including complications and quality of life) will be published separately.

### Definitions

The pancreatic and periampullary region is defined as the pylorus, the duodenum, the pancreatic head, and the distal bile duct. Complications are classified using the Clavien–Dindo score [21]. Major complications are defined as Clavien–Dindo score III or higher. Pancreatic fistula [22, 23], chyle leakage [24], delayed gastric emptying [25], and postpancreatectomy hemorrhage [26] are classified using the International Study Group on Pancreatic Surgery definitions. Bile leak is scored using the International Study Group on Liver Surgery definition [27]. Surgical site infection is classified according to the Centers for Disease Control and Prevention definition [28]. In the case of malignancy, resection margins, including transectional and circumferential margins, are classified as R0 (distance margin to tumor > 1 mm), R1 (distance margin to tumor ≤ 1 mm) and R2 (macroscopically tumor positive margin) [29]. The TNM stage will be classified according to the American Joint Committee on Cancer classification, 7th and 8th editions.

### Quality and safety

All participating surgeons have at least 5 years of experience in open pancreatoduodenectomy. Surgeons who have completed LAELAPS training in minimally invasive distal pancreatectomy and LAELAPS-2 training in MIPD, and who have performed at least 20 MIPDs can participate in the LEOPARD-2 trial. For robot-assisted pancreatoduodenectomies, surgeons will have completed the LAELAPS-3 training program, consisting of the da Vinci® training course, video training, bio tissue training, and proctoring on-site according to the Pittsburgh approach. Surgeons are considered to have passed the learning curve for safety parameters associated with MIPD after performing 20 MIPDs (excluding procedures with open reconstruction), as confirmed by a recent worldwide survey [5]. To obtain a high quality of surgical care in the LEOPARD-2 trial, the protocol committee developed six quality criteria that must be met to allow participation in the trial. All participating surgeons will have to meet the following criteria:

- Performed ≥ 50 advanced minimally invasive gastrointestinal procedures (Advanced laparoscopic gastrointestinal procedure: defined arbitrarily as any laparoscopic gastrointestinal procedure beyond diagnostic laparoscopy, cholecystectomy, and appendectomy.)
- Completed LAELAPS training in minimally invasive distal pancreatectomy
- Completed LAELAPS-2 training in MIPD
- Performed ≥ 50 pancreatoduodenectomies (either MIPD or open pancreatoduodenectomy)
- Performed ≥ 20 MIPDs
- Employed in a center in which ≥ 10 MIPDs and ≥ 20 pancreatoduodenectomies (minimally invasive and open) are performed annually (This cut-off for MIPD is based on a large registry study from the USA [30]. The cut-off for all pancreatoduodenectomies is similar to the current guideline of the Dutch surgical society.)

Surgical videos of procedures performed within the MIPD group will be collected. Technical quality will be assessed at the end of the study by an independent expert. All (serious) adverse events up to 3 months postoperatively will be recorded. Only serious adverse events will be reported through a web portal to the central committee on research involving human subjects (in Dutch: Centrale Commissie Mensgebonden Onderzoek) and the accredited institutional review board (www.toetsingonline.nl). This includes all serious adverse events that necessitate intensive care unit admission, surgical intervention, or readmission, or result in (all-cause) mortality. The remaining events are recorded in an annual overview list. An independent data safety monitoring board will discuss safety parameters after 25, 40 (i.e. at completion of phase 2), 80, and 110 inclusions. The data safety monitoring board exists of an independent statistician or epidemiologist, an independent gastroenterologist, and an independent surgeon (who will chair the board). The advice of the data safety monitoring board meeting will be shared with the steering committee and the sponsor of the trial.

### Go/no-go decision

After completion of phase 2, the results will be discussed in a meeting of the data safety monitoring board and the protocol committee. This moment is considered the “go/no-go” decision. Phase 3 of the LEOPARD-2 trial will only follow when the data safety monitoring board and the protocol committee have no concerns on patient safety. The conclusions of the data safety monitoring board and the protocol committee will be sent to the medical ethics review committee.

### Statistical aspects

#### Sample size calculation

The LEOPARD-2 trial is designed as a phase 2/3 superiority trial, hypothesizing that the postoperative time to functional recovery is shorter after minimally invasive than open pancreatoduodenectomy. Based on a systematic review and meta-analysis [4], a time to functional recovery of 14 days in the control group (open pancreatoduodenectomy) versus 11 days in the intervention group (MIPD) is expected, with a standard deviation of 5 days. Significance level (*α*) is set at 0.05 and power (1 − *β*) at 80%. Including 10% of cross-over from MIPD to open pancreatoduodenectomy, 2% loss to follow-up rate (based on previous surgical trials in the Netherlands), 10% of patients with metastasized disease (included, but oversampled to allow sufficiently powered per-protocol analyses), and after dividing by the asymptotic relative efficiency parameter of the Mann–Whitney U test (0.955) because of expected non-normally distributed data, 68 patients will be randomized in each group. Thus, in total 136 patients will be randomized within the LEOPARD-2 trial.

### Statistical analysis

Primary and secondary outcomes will be cross-checked with data from primary sources and a blinded adjudication committee (blinded for treatment allocation) will check them against the definitions, which were established before the start of this trial. Categorical variables will be compared using the chi-square or Fisher’s exact test as appropriate, and values will be expressed as proportions with corresponding risk ratios and 95% confidence intervals. The distribution of continuous variables will be determined using visual inspection and the Kolmogorov–Smirnov test. For comparison of normally distributed continuous variables the independent-samples *t*-test will be used and values will be expressed as means with standard deviations. Continuous non-normally distributed variables will be compared using the Mann–Whitney U test and values will be expressed as medians with interquartile ranges.

The primary endpoint is a time-to-event endpoint. However, considering that (a) the duration of recovery will most probably be censored in only 4% of patients at a maximum and that (b) taking the full length of the postoperative observation period as a proxy estimate for the time to recovery in these outlying patients will hardly affect the comparison of the study groups because of the non-parametric testing strategy with the Mann–Whitney U test and, finally, that (c) a power of 80% for a Kaplan–Meier analysis as its best alternative can only be achieved at the cost of a much higher and infeasible patient inclusion rate, we will analyze the data as if no censoring takes place. Additionally, the percentage of patients per study arm who do not recover during the observation period will be reported as a secondary outcome measure.

A difference with a two-tailed *P*-value < 0.05 will be considered statistically significant. A multivariable linear regression model will be used to assess potential differences in primary outcome between groups in the presence of potentially confounding factors. Linear mixed modeling will be applied to estimate differences between groups in successive EQ-5D-5 L and QLQ-C30 assessments over time. For exploratory purposes, a secondary analysis will be performed, comparing outcomes for patients with pancreatic ductal adenocarcinoma versus other disease, comparing completed minimally invasive (i.e. no conversion) versus open pancreatoduodenectomy and comparing time to functional recovery between minimally invasive and open pancreatoduodenectomy in complicated (Clavien–Dindo grade III or higher complication) and uncomplicated cases and for the impact of robot-assisted versus laparoscopic pancreatoduodenectomy. A non-inferiority analysis will be performed for Clavien–Dindo grade III or higher complications. The study has an 80% power (*α* = 0.05) to confirm non-inferiority of minimally invasive versus open pancreatoduodenectomy, when 15% fewer patients experience a clinically relevant complication in the intervention group than the control group (non-inferiority margin set at 8%). In the intervention group, robot-assisted procedures may be performed. The amount of robot-assisted procedures is expected to be less than 20%; these will be analyzed separately during cost-analysis.

### Premature termination of the study

After the start of LEOPARD-2 phase 3, no interim analysis will be performed, since this is the first multicenter trial on this topic and any outcome would add to the existing knowledge and be relevant for clinicians worldwide. Also, no stopping rule for superiority will be used since the number of expected events is too small. An independent, unblinded data safety monitoring board will analyze safety end points and may advise the trial steering committee to adjust or stop the study, or to perform a full interim analysis.

### Dissemination policy

The results of the LEOPARD-2 trial will be submitted to a peer-reviewed journal regardless of the study outcome. Authorship will be based on international guidelines. Participants who do not fulfill the authorship criteria will be listed as “collaborator”.

## Discussion

The LEOPARD-2 trial is a multicenter randomized controlled phase 2/3 trial designed to assess whether MIPD is associated with a shorter time to functional recovery than open pancreatoduodenectomy. LEOPARD-2 follows the LEOPARD trial on minimally invasive versus open distal pancreatectomy and the Longitudinal Assessment and Realization of Minimally Invasive Distal Pancreatectomy in the Netherlands training programs (LAELAPS-1, −2 and −3) in minimally invasive distal pancreatectomy, laparoscopic, and robot-assisted pancreatoduodenectomy [11, 12]. These programs were initiated by the DPCG, a national collaboration of surgeons, gastroenterologists, medical oncologists, pathologists, (interventional) radiologists, dietitians, and nurses.

All randomized controlled trials on new surgical techniques face a dilemma over when to start including patients. During the design phase of the LEOPARD-2 trial, the timing of the start of the trial in relation to the learning curve was discussed at several meetings. Previous surgical trials on minimally invasive surgery have been criticized for their timing, because of either starting too early or too late. For instance, the multicenter, randomized controlled MANCHET trial on minimally invasive fundoplication in gastro-esophageal reflux disease was criticized for starting too early (i.e. before completion of the learning curve) with consequently inferior results for the minimally invasive approach [31, 32]. This was confirmed by a follow-up study (i.e. after the learning curve) by the same group, which found superior outcomes for minimally invasive fundoplication. Recently, the ORANGE trial on minimally invasive liver surgery was stopped early because of slow accrual owing to strong patient and surgeon preference for the minimally invasive approach [19]. One could conclude that this trial was initiated too late, in a phase when both patients and surgeons felt the minimally invasive approach was shown to be superior, and the essential period of perceived surgical equipoise had already been passed.

The DPCG initiated a structured training program for minimally invasive pancreatic surgery in the Netherlands. After training in minimally invasive distal pancreatectomy (LAELAPS-1), outcomes were superior as compared with the period prior to training, including 8% conversion rate and 0% mortality in 130 minimally invasive procedures. Consequently, the same teams of surgeons were trained in laparoscopic pancreatoduodenectomy (LAELAPS-2). Again, outcomes were sufficient, including 11% conversion and 4% complication-related mortality in the first 114 procedures from four centers who completed ≥ 20 MIPDs [12]. Next, the length of the learning curve was discussed, taking the argument on timing (not too early, not too late) into account. Based on the results for the first 114 procedures and a previous report, a minimum of 20 total minimally invasive procedures was decided to be the cut-off to participate in the LEOPARD-2 trial [33].

This cut-off of 20 procedures was also reported in an international survey among 435 pancreatic surgeons [5]. Possibly, better outcomes could be obtained with higher cut-offs, such as 80 procedures as reported in a study for operative time, but not for conversion or mortality, where 20 procedures seemed to suffice [34]. However, as this study does not focus on operative time as the primary endpoint measure and such a high cut-off might lead to the danger of performing the trial too late, leading to passing the critical period of equipoise and both the patient’s and doctor’s preference shifting to some extent, postponing the trial would be fatal for its execution. Furthermore, it should be realized that it will take 4 to 8 years for most centers to perform 80 procedures minimally invasively. It would seem unethical if so many patients were to be exposed to a potentially increased surgical risk during such a long learning period. In other words, the cut-off of 20 procedures was also chosen for pragmatic reasons, including high external validity (i.e. reflecting clinical practice in most centers), rather than for the highest likelihood of positive results (i.e. superior outcomes for MIPD).

Another point of discussion is the primary endpoint. As in the LEOPARD-1 [14] and ORANGE trials [19, 20], postoperative time to functional recovery was chosen as the most objective parameter for postoperative recovery. Length of hospital stay was considered less objective, as it is also related to patient preference, home care facilities, and doctor preferences. An alternative endpoint measure, overall or clinically relevant complications, has mainly been reported in retrospective studies and a significant difference between minimally invasive and open pancreatoduodenectomy has not been confirmed by case-matched studies or meta-analysis [3, 4]. Both complications and length of hospital stay are, however, assessed in the LEOPARD-2 trial and will be reported in the final publication.

Because of the standardized reporting of the LAELAPS-1 and −2 training programs, the LEOPARD-1 and −2 trial registration, the LEOPARD-1 and −2 study protocols and final publication of results, the internal validity can be well interpreted. The LEOPARD-2 trial includes all adult patients undergoing elective pancreatoduodenectomy for nearly all indications. Additionally, surgical procedures will be performed in both academic hospitals and large teaching hospitals by surgeons who have all completed the LAELAPS-1 and −2 training programs. This should be taking into account when assessing the external validity of LEOPARD-2. Our group strongly recommends this approach of specific procedure-based training before starting MIPD, given the obvious complexity of the procedure with the required surgical skills.

Blinding of patients in surgical trials has long been deemed impossible. However, several randomized controlled surgical trials have shown that patient blinding is feasible in case of a dedicated protocol committee and trial coordination [14, 19, 20]. Multiple publications in high-impact journals have emphasized the importance of blinding in randomized studies, as in surgical studies results are also likely to be influenced by the patient’s expectation (i.e. the Hawthorne effect) [35]. As in the LEOPARD trial, we attempt to improve study quality as much as possible, and therefore decided to add patient blinding. In this way, we minimize the effect of patient’s expectation on the primary study outcome measure, time to functional recovery, an endpoint measure that is obviously influenced by psychological factors.

In conclusion, the LEOPARD-2 trial is a multicenter randomized controlled phase 2/3, superiority trial investigating safety and time to functional recovery of MIPD and open pancreatoduodenectomy performed by surgeons who have performed ≥ 20 MIPDs within a dedicated training program. This trial aims to provide level one evidence on the added value of the minimally invasive approach in daily practice within high-volume (>20 pancreatoduodenectomies annually) centers, just after 20 procedures have been performed. When this hypothesis is confirmed, it will enhance the worldwide implementation of MIPD, and consequently improve overall patient outcome.

### Trial status

The first patient was randomized on 2 March 2016. LEOPARD-2 phase 2 has been completed and we continued with LEOPARD-2 phase 3. At the time of protocol submission (September 2017), four centers were actively recruiting patients for the trial, two centers were about to start recruiting, and 101 of 136 patients (74%) had been randomized. Inclusion is according to schedule.

## Additional file

Additional file 1: SPIRIT 2013 checklist. (DOCX 49 kb)

## Abbreviations

DPCG: Dutch Pancreatic Cancer Group
EQ-5D-5 L: Euro-QoL five health dimensions questionnaire
ERAS®: Enhanced recovery after surgery
IDEAL: Idea, Development, Exploration, Assessment, and Long-term
LAELAPS: Longitudinal Assessment and Realization of Minimally Invasive Distal Pancreatectomy in the Netherlands
LEOPARD: Minimally invasive versus open distal pancreatectomy
MIPD: Minimally invasive pancreatoduodenectomy
OPD: Open pancreatoduodenectomy
QLQ-C30: Quality of life questionnaire including 30 questions
SPIRIT: Standard Protocol Items: Recommendations for Interventional Trials
TNM: Tumor, node, metastasis.
